# Assessment of Measurement Uncertainty for S-Parameter Measurement Based on Covariance Matrix

**DOI:** 10.3390/s24113668

**Published:** 2024-06-05

**Authors:** Jiangmiao Zhu, Yifan Wang, Kejia Zhao, Yidi Wang, Chaoxian Fu, Kaige Man

**Affiliations:** 1 Faculty of Information Technology, Beijing University of Technology, Beijing 100124, China; zhujiangmiao@bjut.edu.cn (J.Z.); 18301192013@163.com (Y.W.); 18801002896@163.com (Y.W.); 17337691790@163.com (C.F.); 13911778235@163.com (K.M.); 2 National Institute of Metrology, Beijing 100029, China

**Keywords:** measurement uncertainty, covariance matrix, vector network analyzer (VNA), S-parameters

## Abstract

S-parameters are widely used to detail the scattering parameters of radio frequency (RF) components and microwave circuit modules. The vector network analyzer (VNA) is the most commonly used device for measuring S-parameters. Given the multiple frequency points, complex values, and intricate uncertainty propagation involved, accurately assessing the uncertainty of S-parameter measurements is difficult. In this study, we proposed a new method for assessing S-parameter uncertainty based on the covariance matrices, tracing back to the nominal uncertainty of calibration standards. First, we analyzed the relevant theory of uncertainty assessment using covariance matrices and subsequently deduced the mechanism of Type B uncertainty propagating from calibration standards to error model coefficients and S-parameter measurements to evaluate Type B measurement uncertainty. In this study, a novel measurement system was constructed for measuring grounded coplanar waveguides by using a VNA and calibration standards with 8- and 12-error models. Initially, the model assessed the Type B uncertainty of measuring four S-parameters of a grounded coplanar waveguide. Next, the VNA calibrated with the 12-error model was used to conduct multiple repeated measurements to assess the Type A uncertainty of the grounded coplanar waveguide. Finally, the composite uncertainty was constructed, which demonstrated that the proposed method can be used for assessing the uncertainty of S-parameters.

## 1. Introduction

With the rapid development of electronic communication technology, scattering parameters (S-parameters) can provide accurate characteristics of electromagnetic wave transmission in microwave components and circuit network modules. S-parameters are network parameters that are established based on the relationship between incident waves and reflected waves, and the accuracy of their measurement results directly affects the quality of microwave components. The vector network analyzer (VNA) is the most widely used equipment for measuring S-parameters. The measurement uncertainty of the VNA considerably influences the uncertainty of S-parameters and should be evaluated as a Type B uncertainty.

Measurement uncertainty is a scientific evaluation indicator for measurement results. Common methods for assessing uncertainty generally involve the law of propagation of uncertainty based on measurement uncertainty representation guidelines and the Monte Carlo method based on mathematical modeling [1,2]. However, for assessing the complex uncertainty of S-parameters involving multiple frequency points and variables, the covariance matrix method is preferred. The covariance matrix is considered an extension of linear propagation, which can effectively address the uncertainty evaluation and propagation of such complex objects [3,4,5,6].

Numerous studies have focused on the measurement uncertainty of VNA. However, most methods are limited to assessing Type A uncertainty of the measurement results of S-parameters and do not fully consider the influence of the instrument on measurement uncertainty. Type B uncertainty is not considered because of the difficulty in evaluation. Type B uncertainty has complex influencing factors and numerous unmeasurable intermediate quantities in the equipment that are typically ignored. Type A uncertainty is typically the uncertainty introduced by the randomness of measurements, which is generally assessed through multiple repeated measurements. Type B uncertainty, on the other hand, is the inherent uncertainty of the measurement system itself, requiring an in-depth analysis of the sources of uncertainty and the assessment methods [2]. Since the proposal of the 8-term and 12-term calibration error models in the 1970s [7], many scholars have conducted extensive research on sources of error in VNA measurements, the impact of various calibration error models on measurement results, and the relationship between calibration standards and error model coefficients, focusing on VNAs with bandwidths lower than 30 GHz and tens of sampling points [8,9,10,11,12]. Because the transmission bandwidth frequency reaches the terahertz level, higher requirements have been proposed for the bandwidth and number of sampling points in VNA uncertainty assessment, which is crucial for the subsequent propagation of S-parameter uncertainty [13,14].

This paper proposes a method to evaluate the uncertainty of VNA-measured S-parameters based on the covariance matrix. Using the 8-term and 12-term error models of dual-port calibration, the uncertainty is progressively propagated to the measured S-parameters. Additionally, our algorithm is suitable for more complex scenarios with higher bandwidths and more frequency points. To consider the multi-frequency measurement of VNA and the correlation between variables and facilitate the propagation of S-parameter uncertainty, the measurement uncertainty of VNA was evaluated using the covariance matrix. Experimental design schemes and formula derivations have been provided, and the same VNA is calibrated using 8- and 12-term error models. Experimental verification of Type B uncertainty assessment was performed by measuring the S-parameters of the ground coplanar waveguide. Finally, Type A uncertainty was assessed multiple times based on calibration with the 12-term error model, and the combined uncertainty was calculated.

## 2. Covariance Matrix Uncertainty Assessment

For complex measurement results, the covariance matrix is widely used in the uncertainty assessment method. When assessing the Type A uncertainty of multivariate measurements, the covariance of the off-diagonal elements can effectively retain the correlations between variables. However, when assessing Type B uncertainty, because of the complexity of uncertainty sources in the measurement system, tracing back to the final source requires multiple passes. Covariance matrix operations have numerous advantages; for example, the correlation between variables in the previous step affects the uncertainty of the covariance matrix in the next step, rendering such assessments scientifically reliable [15,16]. Although the law of propagation of uncertainty can be used to separately calculate the effect of correlations, in complex multi-input, multi-output propagation models, using the covariance matrix is highly convenient [17].

The covariance matrix is a semi-positive definite matrix. For a covariance matrix constructed for random variables, shown in Equation (1), the elements on the diagonal represent the variance of each random variable, and the off-diagonal elements represent the covariance of the corresponding random variables. Thus, the influence of correlations can be reflected by performing uncertainty propagation calculations as follows:

(1) $${\sum =\left[\begin{array}{c} \sigma (x_{1},x_{1})\\ \ldots \ldots \\ \sigma (x_{d},x_{1}) \end{array}\ldots \ldots \begin{array}{c} \sigma (x_{1},x_{d})\\ \ldots \ldots \\ \sigma (x_{d},x_{d}) \end{array}\right]}_{d\times d}$$

Each element in Equation (1) can be determined by Equation (2); the covariance between the m-th and k-th random variables can be expressed as:

(2) $$\sigma \left(x_{m},x_{k}\right)=\frac{1}{n-1}\sum_{i=1}^{n}(x_{mi}-{\bar{x}}_{m})(x_{ki}-{\bar{x}}_{k})$$

Here, i represents the i-th measurement value of a random variable.

Suppose a random variable **X** of dimension m × 1 with a mean $X_{0}$, and a functional relationship exists such that Y = f(X) between vector **Y**(n × 1) and X, where *f* is a continuously differentiable function near $X_{0}$. Next, f (X) can be linearly approximated around $X_{0}$ as given in Equation (3):

(3) $$Y=f\left(X\right)\approx f\left(X_{0}\right)+J\cdot \left(X-X_{0}\right)+L,$$

where L represents the higher-order remainder term of the Taylor expansion. When analyzing specific problems, in most cases, the first-order expansion is sufficient, but sometimes checking whether the higher-order remainder term can be ignored (without affecting the overall result). Here, J is the Jacobian matrix, where each element represents the sensitivity coefficient for uncertainty propagation, calculated as follows:

(4) $$J_{ij}={\left.\frac{\partial F_{i}(X)}{\partial x_{j}}\right|}_{x_{0}}={\left.\frac{\partial y_{i}}{\partial x_{j}}\right|}_{x_{0}}$$

Based on Equations (3) and (4), the covariance matrix relationship between random variables X and Y can be obtained as follows:

(5) $$\sum Y=E\left[\left(Y-Y_{0}\right)\left(Y-Y_{0}\right)*\right]\approx E\left[J\left(X-X_{0}\right)\left(J\left(X-X_{0}\right)\right)*\right]\approx J\cdot \sum X\cdot J*\;.$$

The uncertainty covariance matrix of Y can be obtained using Equations (1)–(5). However, in many cases, the functional relationship between random variables is a complex implicit relationship that sometimes cannot be described using mathematical expressions. In such cases, numerical methods such as finite difference methods can be used to approximate the Jacobian matrix.

The combined uncertainty of measurement results includes Type A uncertainty and Type B uncertainty, as presented in Equation (6). For each measurement, a covariance matrix $\sum S_{k}$, is constructed to describe the Type B uncertainty of that measurement. After K measurements, a covariance matrix ∑r is constructed to describe the overall Type A uncertainty of K measurements.

With the increase in the number of measurements K, Type A uncertainty decreases, whereas Type B uncertainty remains unchanged, becoming the dominant component in the combined uncertainty as follows:

(6) $$\sum U=\frac{1}{K}\sum_{k=1}^{K}(\sum S_{k})+\frac{1}{K}\sum r.$$

## 3. VNA Measurement Uncertainty Analysis

Calibration is necessary when measuring S-parameters with a VNA because of the presence of an error network between its measurement plane and the device under the test’s measurement plane. Calibration involves obtaining error model coefficients, which can be used to correct the directly measured results of the VNA and obtain accurate measurement results. Therefore, when analyzing the uncertainty of S-parameter measurements, the influence of the error model coefficients obtained through calibration on the final results should be considered, and the uncertainty of the error model coefficients can be traced back to the uncertainty of the calibration standards.

**Figure 1:**
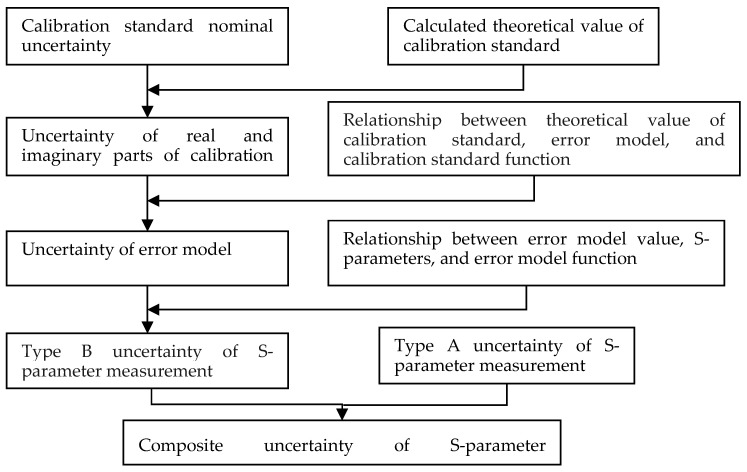
Flowchart of uncertainty propagation in VNA measurement of S-parameters.

The process of measuring S-parameters with a VNA is a complex computational process, where uncertainty is progressively propagated according to the computational process, from the calibration standards to the error model coefficients and subsequently to the measured values of S-parameters. Figure 1 illustrates the flow of uncertainty propagation.

S-parameters are complex functions of frequency. When assessing the uncertainty of S-parameter measurements point by point, considering the correlation between frequency points and parameters as well as the stepwise propagation process, a covariance matrix can accurately describe this problem [10].

## 4. VNA Measurement Type B Uncertainty Assessment Process

### 4.1. Uncertainty Analysis of Calibration Standards

VNA calibration requires three calibration standards: short, open, and load. In two-port calibration, a thorough standard is also required. The manufacturer provides the corresponding nominal uncertainties for the calibration standards. Typically, the uncertainties of the short and open standards originate from phase errors, whereas the uncertainty of the load standard is given by the range of true values specified by the manager.

The corresponding error model coefficients can be obtained by analyzing the relationship between the measured values and the true values of the calibration standards before VNA correction. Ideally, the complex reflection coefficients of the three calibration standards are: $\Gamma_{short}=-1,\Gamma_{open}=1,\Gamma_{load}=0$. However, in practice, the true values of manufactured calibration standards deviate from the ideal values. A circuit model can be established based on their characteristics to calculate the true values.

For short-circuit calibration standards within hundreds of gigahertz, the values exhibit inductive characteristics. Their approximate circuit model is a frequency-dependent third-order curve fitting model:

(7) $$\left\{\begin{array}{c} L\left(f\right)=L_{0}+L_{1}\cdot f+L_{2}\cdot f^{2}+L_{3}\cdot f^{3}\\ \Gamma_{short}=\frac{j2\pi fL\left(f\right)-Z_{0}}{j2\pi fL\left(f\right)+Z_{0}}e^{-j4\pi L/\lambda } \end{array}\right..$$

In Equation (7), $L_{0}-L_{3}$ represent the inductance parameter related to frequency, provided by the calibration device manufacturer. The specific parameters used in this paper are shown in Table 1 and Table 2, the specific parameters are provided by the calibration manufacturer. λ represents the wavelength of the electromagnetic wave, and f represents the frequency, $Z_{0}$ represents the characteristic impedance value of the calibration standard, L represents the electrical length between the reference plane and the actual short circuit plane. Thus, the reflection coefficient at the reference plane is $\Gamma_{0}=e^{-4\pi Lj/\lambda }$ because of the existence of a length of transmission line between the real short circuit plane and the connection point with the VNA. Therefore, the complex reflection coefficient of the short circuit standard, $\Gamma_{short}$ can be expressed by Equation (7).

Similarly, open-circuit components exhibit capacitive characteristics, with their circuit model and complex reflection coefficient represented as follows:

(8) $$\left\{\begin{array}{c} C\left(f\right)=C_{0}+C_{1}\cdot f+C_{2}\cdot f^{2}+C_{3}\cdot f^{3}\\ \Gamma_{open}=\frac{1-j2\pi fC\left(f\right)Z_{0}}{1+j2\pi fC\left(f\right)Z_{0}}e^{-j4\pi L/\lambda } \end{array}\right..$$

The load component exhibits a resistance characteristic that is used to provide impedance matching within the measurement frequency band with the system. The performance of the component deteriorates with the increase in frequency, and the manufacturer provides the circuit loss of the load calibration component, typically in the range of several tens of dB, which can be used as the maximum uncertainty.

In dual-port calibration, a through calibration component is required, such as the most common short-load-open-through calibration, based on four fully known calibration components. Additionally, an unknown through calibration method can be used, adhering to the reciprocity condition S12 = S21 of the through calibration component in dual-port measurements. In this study, the method of unknown through calibration is used, and the uncertainty introduced by the through calibration component is ignored.

Because the S-parameters are complex, when assessing uncertainty, they should be divided into real and imaginary parts, rendering storage and computation easy. Typically, the nominal uncertainty of calibration components originates from phase; therefore, the nominal uncertainty of calibration components should be transferred to the uncertainty of their real and imaginary parts first. For nominal uncertainty, the correlation between various frequency points is not considered, and the covariance matrix of the nominal uncertainty of each calibration component is a diagonal matrix. Considering the open-circuit component as an example, the process of transferring the nominal uncertainty to the uncertainty of its real and imaginary parts is demonstrated. The relationship between real and imaginary parts given by Equation (9) and the calculation method of sensitivity coefficients to amplitude and phase are used to construct the required Jacobian matrix $J_{\Gamma_{o}}$, as shown in Equation (10):

(9) ReΓo=Γocos⁡ΦΓoImΓo=Γosin⁡ΦΓo∂ReΓo∂ΦΓ0=−ImΓo ∂ImΓo∂ΦΓo=ReΓo,

(10) JΓo=∂ReΓo∂ΦΓ0∂ImΓo∂ΦΓo=−ImΓoReΓo.

Such a Jacobian matrix exists at each frequency point. According to the uncertainty transfer process of Equation (5), the uncertainty covariance matrix of the real and imaginary parts of the open-circuit component can be obtained through Equation (11). In this matrix, the elements on the main diagonal are the squares of the uncertainties of the real and imaginary parts of the open-circuit component, and the off-diagonal elements are their covariance.

(11) $$\sum \Gamma_{o}=J_{\Gamma_{o}}\sum \Phi_{\Gamma_{o}}{J_{\Gamma_{o}}}^{T}$$

Similarly, the covariance matrix of the short circuit component and the load component can be obtained. At each frequency point, the covariance matrices of the short-circuit, open-circuit, and load components are concatenated into a block diagonal matrix to obtain the overall covariance matrix of the calibration components.

### 4.2. Uncertainty Analysis of Error Models

#### 4.2.1. Single-Port Measurement

**Figure 2:**
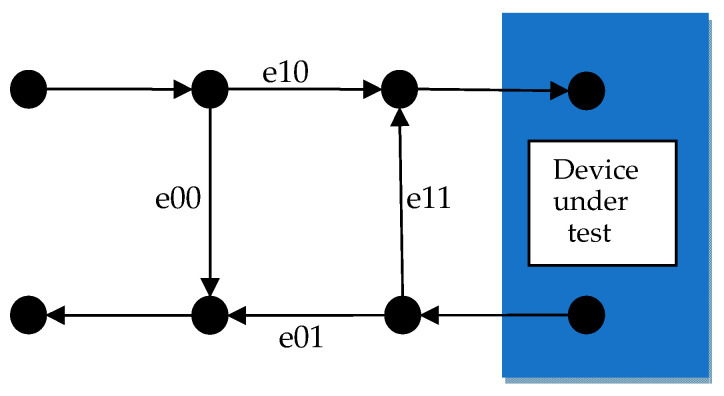
Schematic of the three-error model for single-port measurements.

The error model network used for single-port calibration consists of three error models [12] (Figure 2):

When conducting single-port measurements, the relationship between the reflection coefficient $\Gamma_{DUT}$ of the device under test (DUT) and its measured value $\Gamma_{M}$ is given by Equation (12), where $e_{00},e_{11},$ and $∆e=e_{00}e_{11}–e_{01}e_{10}$ represent the directional error, source match error, and frequency response error, respectively. Based on calibration-derived error model coefficients, a VNA can correct the raw measurement values of the device to obtain measurement results closer to the true values as follows:

(12) $$\Gamma_{DUT}=\frac{\Gamma_{M}-e_{00}}{\Gamma_{M}e_{11}-\Delta e}$$

The calibration process follows Equation (12). During VNA calibration, the calibration kit type used is first selected, and the calibration process is similar to measuring the calibration kit with the VNA. By comparing the measured values of the calibration kit with their true values, the error model coefficients $e_{00},e_{11},$ and ∆e are inversely solved to establish the functional relationship between the three types of calibration kits as follows:

(13) $$\left\{\begin{array}{c} e_{00}=\frac{\Gamma_{O}\Gamma_{MO}\left(\Gamma_{MS}\Gamma_{L}-\Gamma_{ML}\Gamma_{S}\right)+\Gamma_{S}\Gamma_{MS}\left(\Gamma_{ML}\Gamma_{O}-\Gamma_{MO}\Gamma_{L}\right)+\Gamma_{L}\Gamma_{ML}(\Gamma_{MO}\Gamma_{O}-\Gamma_{MS}\Gamma_{O})}{\Gamma_{O}\Gamma_{MO}\left(\Gamma_{L}-\Gamma_{S}\right)+\Gamma_{S}\Gamma_{MS}\left(\Gamma_{O}-\Gamma_{L}\right)+\Gamma_{L}\Gamma_{ML}(\Gamma_{S}-\Gamma_{O})}\;;\\ e_{11}=\frac{\Gamma_{MO}\left(\Gamma_{L}-\Gamma_{S}\right)+\Gamma_{MS}\left(\Gamma_{O}-\Gamma_{L}\right)+\Gamma_{ML}(\Gamma_{S}-\Gamma_{O})}{\Gamma_{O}\Gamma_{MO}\left(\Gamma_{L}-\Gamma_{S}\right)+\Gamma_{S}\Gamma_{MS}\left(\Gamma_{O}-\Gamma_{L}\right)+\Gamma_{L}\Gamma_{ML}(\Gamma_{S}-\Gamma_{O})}\;;\\ \Delta e=\frac{\Gamma_{O}\Gamma_{MO}\left(\Gamma_{MS}-\Gamma_{ML}\right)+\Gamma_{S}\Gamma_{MS}\left(\Gamma_{ML}-\Gamma_{MO}\right)+\Gamma_{L}\Gamma_{ML}(\Gamma_{MO}-\Gamma_{MS})}{\Gamma_{O}\Gamma_{MO}\left(\Gamma_{L}-\Gamma_{S}\right)+\Gamma_{S}\Gamma_{MS}\left(\Gamma_{O}-\Gamma_{L}\right)+\Gamma_{L}\Gamma_{ML}(\Gamma_{S}-\Gamma_{O})}\;. \end{array}\right.$$

In Equation (13), $\Gamma_{O},\Gamma_{L},\Gamma_{S}$ are the true values of the calibration kits obtained through calculation and $\Gamma_{MO},\Gamma_{ML},\Gamma_{MS}$ are the measured values of the calibration kits obtained from pre-calibration VNA measurements. Based on this functional relationship and Equation (4), the Jacobian matrix $J_{E3}$ is constructed by taking the partial derivatives of the true values of the calibration standards with respect to each error model coefficient:

(14) $${J_{E3}=\left[\begin{array}{c} \frac{\partial re(e_{00})}{\partial re(\Gamma_{S})}\\ \frac{\partial im(e_{00})}{\partial re(\Gamma_{S})}\\ \ldots \ldots \\ \frac{\partial im(\Delta e)}{\partial re(\Gamma_{S})} \end{array}\ldots \ldots \begin{array}{c} \frac{\partial re(e_{00})}{\partial im(\Gamma_{L})}\\ \frac{\partial im(e_{00})}{\partial im(\Gamma_{L})}\\ \ldots \ldots \\ \frac{\partial im(\Delta e)}{\partial im(\Gamma_{L})} \end{array}\right]}_{6\times 6}$$

(15) $$\sum E3=J_{E3}\;*\;diag\left(\sum \Gamma_{S},\Gamma_{O},\Gamma_{L}\right)*{J_{E3}}^{\prime }$$

(16) $${\sum E3=\left[\begin{array}{c} \sum re(e00)\\ \mathrm{cov}\left(re\left(e00\right),im\left(e00\right)\right)\\ \ldots \ldots \\ \mathrm{cov}(\mathrm{re}\left(e00\right),\mathrm{im}\left(∆e\right)) \end{array}\ldots \ldots \begin{array}{c} \mathrm{cov}(\mathrm{im}\left(∆e\right),\mathrm{re}\left(e00\right))\\ \mathrm{cov}(\mathrm{im}\left(∆e\right),\mathrm{im}\left(e00\right))\\ \ldots \ldots \\ \sum \left(im(∆e)\right) \end{array}\right]}_{6\times 6}$$

Uncertainties can be propagated to the three-error models using Equations (14) and (15). Similarly, based on Equation (12), the Jacobian matrix $J_{S11}$ of the measurement of the S11 parameter with respect to the three-error model coefficients can be constructed as follows:

(17) $${J_{S11}=\left[\begin{array}{c} \frac{\partial re\left(S11\right)}{\partial re\left(e_{00}\right)}\;\\ \frac{\partial im\left(S11\right)}{\partial re\left(e_{00}\right)} \end{array}\ldots \ldots \begin{array}{c} \frac{\partial re\left(S11\right)}{\partial im\left(\Delta e\right)}\;\\ \frac{\partial im\left(S11\right)}{\partial im\left(\Delta e\right)} \end{array}\right]}_{2\times 6};$$

(18) $$\sum S11=J_{S11}*\sum E3*{J_{S11}}^{\prime }$$

(19) $$\sum S11=\left[\begin{array}{c} \sum re\left(S11\right)\\ \mathrm{cov}(\mathrm{re}\left(S11\right),\mathrm{im}\left(S11\right)) \end{array}\begin{array}{c} \mathrm{cov}(\mathrm{im}\left(S11\right),\mathrm{re}\left(S11\right))\\ \sum im(S11) \end{array}\right]$$

Through Equations (17) and (18), the covariance matrix ∑S11 of S11 can be obtained, as presented in Equation (19). This matrix determines the uncertainty of S11, which can then be propagated to other RF parameters.

#### 4.2.2. Two-Port Measurement

**Figure 3:**
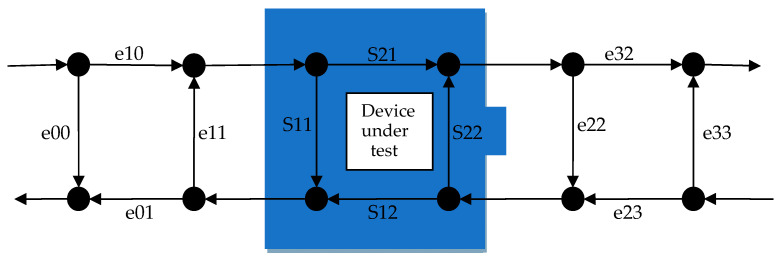
Schematic of the eight-error model for two-port measurements.

**Figure 4:**
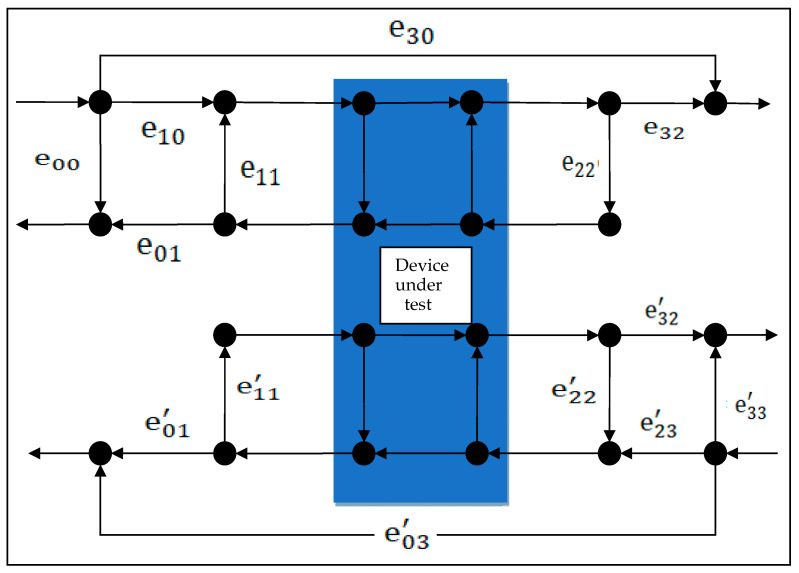
Schematic of a 12-term error model for dual-port measurement.

Although single-port measurements have a simple model, they are seldom used in practice because they can only measure S11 parameters. Most measurements are double-port measurements, but they are based on the foundation of the single-port three-term error model. In the commonly used calibration methods, the 8- or 12-term error models [7], as shown in Figure 3 and Figure 4, are used:

The dual-port 8-term error model can be simplified to seven terms by setting $e_{32}$ to 1; that is, first performing single-port calibration on each of the two single ports to obtain two 3-term error models, and subsequently performing thorough calibration to obtain the seventh term error model ${e_{01}/e}_{32}$, as presented in Equation (20). Among the four S-parameters, S11 and S22 are obtained by single-port measurements at ports A and B, respectively, relying only on two independent 3-term error models, namely S12 and S21, depending on the full 7-term error model.

(20) $$\frac{e_{01}}{e_{32}}=\sqrt{\frac{{S12}_{M}(e_{00}e_{11}-∆e)}{{S21}_{M}(e_{33}e_{22}-{∆e}^{\prime })}}.$$

According to Equation (20), the coefficient of the seventh error model term is related to the coefficients of the previous six-error model terms, as well as to the forward and reverse transmission coefficients of the through a standard. When calibrating the unknown through standard, Equation (20) can be simplified using S12 = S21.

When constructing the Jacobian matrix for the 8-error model, because of the involvement of the collaborative transfer of ${e_{01}/e}_{32}$ and the two 3-error models, a Jacobian matrix should be first constructed to transfer the uncertainties from the coefficients of the first 6-error models (correlation between the three-error models for each port can be ignored) to ${e_{01}/e}_{32}$, and subsequently, synthesize the covariance matrix ${\sum E7}_{14\times 14}$, containing seven-error model coefficients. The Jacobian matrix used for collaborative transfer cannot be expressed in a single mathematical expression. Referring to Equation (14), using two Jacobian matrices is critical to separately calculate the covariance matrix and then concatenate them by blocks.

Because four S-parameters exist in dual-port measurements, storing them in the same covariance matrix facilitates the observation of their correlations. Similar to the method in Section 4.2.1, the Jacobian matrix Equation (21) should be constructed to obtain the covariance matrix ${\sum S}_{8\times 8}$ of S-parameters measurements for dual-port through Equation (22). In this matrix, the primary diagonal stores the uncertainties squared of the real and imaginary parts of S11, S12, S21, and S22, respectively, whereas the off-diagonal elements preserve the correlations between S-parameters.

(21) $$J_{S7}{=\left[\begin{array}{c} \frac{\partial re\left(S11\right)}{\partial re\left(e_{00}\right)}\;\\ \frac{\partial im\left(S11\right)}{\partial re\left(e_{00}\right)}\\ \ldots \ldots \\ \frac{\partial im\left(S22\right)}{\partial re\left(e_{00}\right)} \end{array}\ldots \ldots \begin{array}{c} \frac{\partial re\left(S11\right)}{\partial im\left({e_{01}/e}_{32}\right)}\;\\ \frac{\partial im\left(S11\right)}{\partial im\left({e_{01}/e}_{32}\right)}\\ \ldots \ldots \\ \frac{\partial im\left(S22\right)}{\partial im\left({e_{01}/e}_{32}\right)} \end{array}\right]}_{8\times 14}$$

(22) $$\sum S=J_{S7}*\sum E7*{J_{S7}}^{\prime }$$

In Equation (21), because the parameters S11 and S22 are independent of the seventh error term model ${e_{01}/e}_{32}$, the elements such as $\frac{\partial re\left(S11\right)}{\partial re\left({e_{01}/e}_{32}\right)}$ have a value of 0. However, the parameters S12 and S21 are related to ${e_{01}/e}_{32}$.

As displayed in Figure 4, the 12-term error model is the most commonly used short-load-open-through dual-port calibration method, which involves forward and reverse measurements. For instance, the forward measurement includes directional error $e_{00}$, source match error $e_{11}$, reflection trace error $e_{10}\cdot e_{01}$, transmission trace error $e_{10}\cdot e_{32}$, load match error $e_{22}$, and crosstalk error $e_{30}$. The calibration process similarly starts with single-port calibration for each measurement port separately, obtaining directional error, source match error, and transmission trace error, totaling six terms. Next, a thorough calibration is performed to obtain the remaining error model. Typically, crosstalk error $e_{03}^{\prime }$ and $e_{30}$ can be omitted, and the complete 12-term error model is seldom used, simplifying it to 10 terms.

Based on the signal flow graph, the relationship between the measured S-parameter results according to Equation (23) and the error model coefficients can be obtained as follows [11,18]:

(23) $$\left\{\begin{array}{c} {S11}_{M}=e_{00}+e_{10}e_{01}\frac{S11-e_{22}\Delta S}{1-e_{11}S11-e_{22}S22+e_{11}e_{22}\Delta S}\\ {S12}_{M}=e_{03}^{\prime }+e_{01}^{\prime }e_{23}^{\prime }\frac{S12}{1-e_{11}^{\prime }S11-e_{22}^{\prime }S22+e_{11}^{\prime }e_{22}^{\prime }\Delta S}\\ {S21}_{M}=e_{30}+e_{10}e_{32}\frac{S21}{1-e_{11}S11-e_{22}S22+e_{11}e_{22}\Delta S}\\ {S22}_{M}=e_{33}^{\prime }+e_{32}^{\prime }e_{23}^{\prime }\frac{S22-e_{11}^{\prime }\Delta S}{1-e_{11}^{\prime }S11--e_{22}^{\prime }S22+e_{11}^{\prime }e_{22}^{\prime }\Delta S}\; \end{array}\right.$$

In Equation (23), ${S11}_{M}–{S22}_{M}$ represent the measured values of the device before calibration with the VNA, whereas S11–S22 represent the true values of the device’s S-parameters. Here, ΔS=S11·S22−S12·S21 is similar to the process of the 8-term error model. For obtaining the covariance matrix of the 12-term error model, first, the covariance matrix of some error model coefficients should be obtained. These coefficients should be transmitted to other error model coefficients. This can construct the covariance matrix ${\sum E10}_{20\times 20}$ of the error model coefficients.

By simultaneously solving Equation (23), a system of equations can be solved to obtain the relationship between four S-parameters and their pre-calibration measured values and error model coefficients, as presented in Equation (24). When transmitting the covariance matrix of the error model ∑E10 to the covariance matrix of the S-parameters, the Jacobian matrix ${J_{S10}}_{8\times 20}$ is constructed according to the following Equation and subsequently transmitted to the covariance matrix of the S-parameters. The process is similar to Equations (21) and (22) and is not elaborated here.

(24) $$\left\{\begin{array}{c} S11=(\frac{{S11}_{M}-e_{00}}{e_{10}e_{01}}\left(1+e_{22}^{\prime }\frac{{S22}_{M}-e_{33}^{\prime }}{e_{32}^{\prime }e_{23}^{\prime }}\right)-e_{22}\frac{{S21}_{M}-e_{30}}{e_{10}e_{32}}\frac{{S12}_{M}-e_{03}^{\prime }}{e_{01}^{\prime }e_{23}^{\prime }})/K\\ S12=(\frac{{S12}_{M}-e_{03}^{\prime }}{e_{01}^{\prime }e_{23}^{\prime }}\left(1+\left(e_{11}-e_{11}^{\prime }\right)\frac{{S11}_{M}-e_{00}}{e_{10}e_{01}}\right))/K\\ \;S21=(\frac{{S21}_{M}-e_{30}}{e_{10}e_{32}}\left(1+{(e}_{22}^{\prime }-e_{22}\right)\frac{{S22}_{M}-e_{33}^{\prime }}{e_{32}^{\prime }e_{23}^{\prime }}))/K\\ S22=(\frac{{S22}_{M}-e_{33}^{\prime }}{e_{32}^{\prime }e_{23}^{\prime }}\left(1+e_{11}\frac{{S11}_{M}-e_{00}}{e_{10}e_{01}}\right)-e_{11}^{\prime }\frac{{S21}_{M}-e_{30}}{e_{10}e_{32}}\frac{{S12}_{M}-e_{03}^{\prime }}{e_{01}^{\prime }e_{23}^{\prime }})/K \end{array}\right.$$

Here, $K=\left(1+e_{11}\frac{{S11}_{M}-e_{00}}{e_{10}e_{01}}\right)\left(1+e_{22}^{\prime }\frac{{S22}_{M}-e_{33}^{\prime }}{e_{32}^{\prime }e_{23}^{\prime }}\right)-e_{22}e_{11}^{\prime }\frac{{S21}_{M}-e_{30}}{e_{10}e_{32}}\frac{{S12}_{M}-e_{03}^{\prime }}{e_{01}^{\prime }e_{23}^{\prime }}$.

## 5. Assessment of Uncertainty of S-Parameters of Grounded Coplanar Waveguide

### 5.1. Construction of the Measurement System

**Figure 5:**
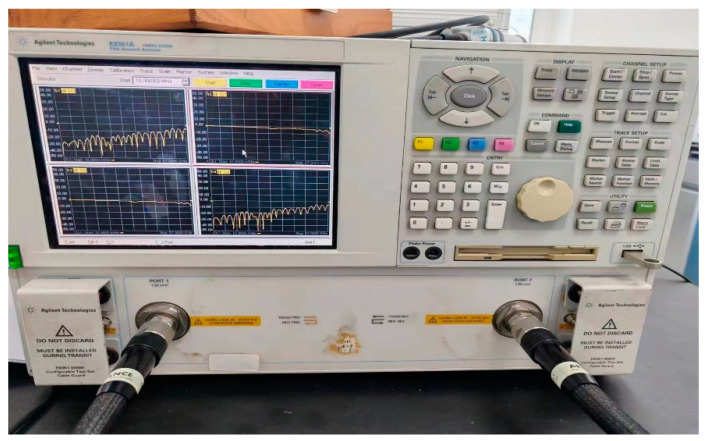
VNA E8361 was used in this study.

**Figure 6:**
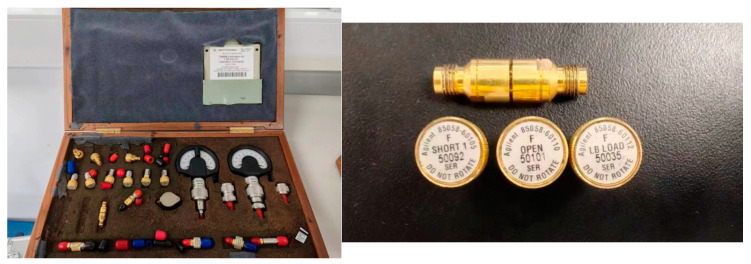
Calibration kit 80858B was used in this study.

In this study, a measurement system was developed for measuring the S-parameters of a grounded coplanar waveguide (GCPW) by using a VNA. The Agilent E8361 VNA was used with its accompanying calibration kit, 85058B, the equipment is all manufactured by Keysight(USA, California). The calibration kit includes SHORT1, OPEN, LB LOAD, and THROUGH (FEMALE). The measurement bandwidth of the VNA was from DC to 67 GHz, and the calibration kit was universal for the frequency band. Other cables and adapters used are disregarded when evaluating uncertainty. Figure 5 and Figure 6 display the physical setup for the measurement.

Table 1 and Table 2 display their nominal uncertainties and related circuit parameters [19,20].

The proposed algorithm sets the input uncertainties for open and short calibration standards based on Table 1, using the return loss of the load standard as the maximum uncertainty in Table 1. The uncertainties and various parameters in the table are provided by the calibration manufacturer. The nominal uncertainty represents the maximum uncertainty of the calibration within this frequency range. In specific experiments, to ensure that the uncertainty deviation of the calibration standards remains within the nominal uncertainty range, the calibration standards should be periodically inspected to ensure reliability. Under favorable conditions, conducting uncertainty assessments on the three calibration standards prior to measurement could yield accurate results.

**Figure 7:**
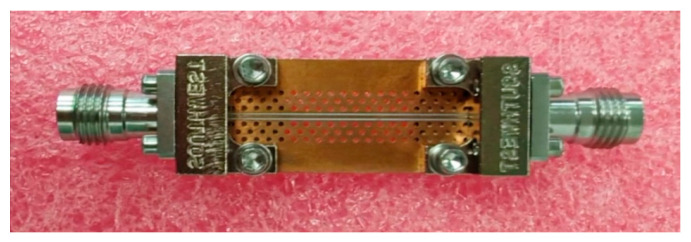
Grounded coplanar waveguide (GCPW) with three-row via array and supported layer.

**Figure 8:**
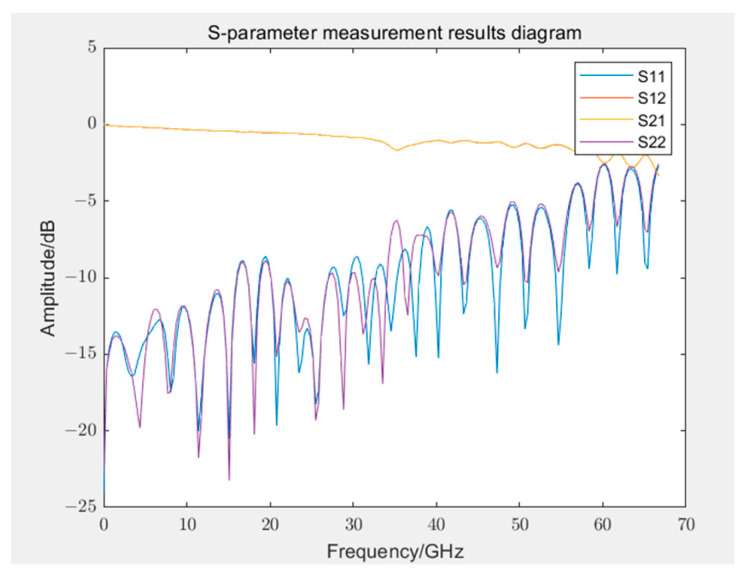
S-parameter measurement results of the GCPW.

The measured GCPW developed independently in the laboratory [21] with a supported-layer, three-row via array had an effective bandwidth of approximately 60 GHz. Although its performance slightly diminishes in the high-frequency range, it does not affect the uncertainty assessment method. Figure 7 depicts the physical representation, and Figure 8 displays the S-parameter measurement results.

In practice, the measurements of three calibration standards are initially conducted without calibration, obtaining the measurement values of the calibration standards. Subsequently, GCPW measurements are performed to yield measurement values for the first four S-parameters before calibration. Next, calibration is performed on the VNA, selecting appropriate calibration standards and error models. On completion of calibration, error model data are extracted from the VNA. Finally, the calibrated VNA was used to measure the GCPW to achieve actual measurement values for the four S-parameters. The experiment sets the initial frequency at 10 MHz, with uniform sampling up to a cutoff frequency of 67 GHz, comprising 1000 frequency points.

In this study, the same calibration standards and VNA are used to measure the GCPW (Figure 7). VNA is calibrated using both 8- and 12-term error models, and a comparison of the uncertainty assessment results is conducted and analyzed.

The method of evaluating uncertainty using the covariance matrix involves multiple iterations, and its complex calculations are time-consuming. The computation can be simplified using the Cauchy–Riemann equations [22] during the complex differentiation stage. For example, the sensitivity coefficient of parameter S11 to the error model coefficient $e_{00}$ is as follows:

(25) $$\left\{\begin{array}{c} \frac{\partial re(S11)}{\partial re(e00)}=\frac{\partial im(S11)}{\partial im(e00)}\\ \frac{\partial re(S11)}{\partial im(e00)}=-\frac{\partial im(S11)}{\partial re(e00)} \end{array}\right.$$

The operations required for constructing the Jacobian matrix can be halved, as presented in Equation (25).

### 5.2. Uncertainty Assessment Results Analysis

**Figure 9:**
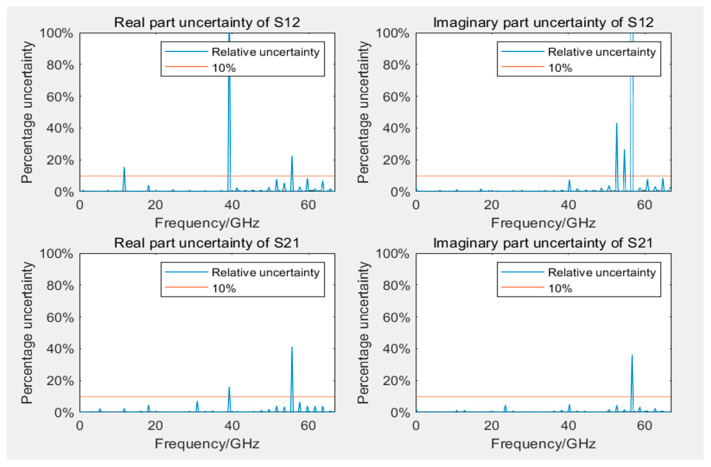
Uncertainty of real and imaginary parts of S12 and S21 in the 12-term error model calibration.

**Figure 10:**
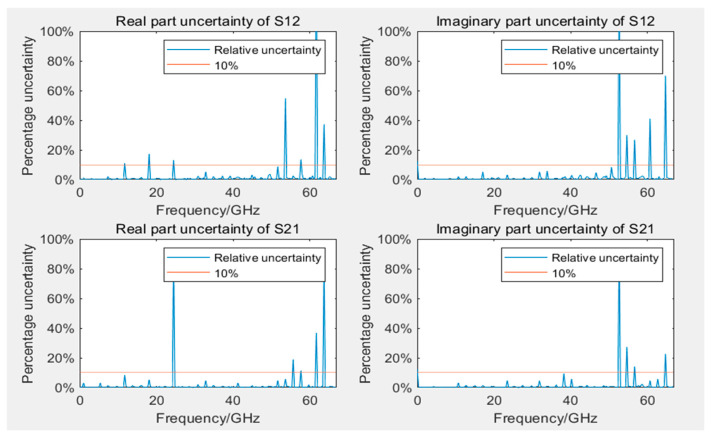
Uncertainty of real and imaginary parts of S12 and S21 in the 8-term error model calibration.

Figure 9 and Figure 10 display the Type B uncertainty of measuring S12 and S21 parameters during the calibration of 12- and 8-term error models. Because of computational complexity, in evaluating Type B uncertainty, the data collected by VNA are resampled at evenly spaced intervals, totaling 200 frequency points. In engineering applications, it is generally accepted that measurement results are considered reliable if the uncertainty is below 10%. The figures reveal that, with the exception of 3–5 outliers among the 200 frequency points, the measurement results at most frequency points were reliable, even if they were lower than 10%. Some outliers had significantly large uncertainty, possibly because of very small measured values at those frequency points. Most outliers occurred in frequency ranges beyond 50 GHz, which was related to the bandwidths of both VNA and GCPW nearing measurement limits, resulting in performance degradation. Additionally, the uncertainty of calibration standards increases with frequency, which may have influenced measurement results. Furthermore, in S12 and S21, outliers typically occurred at the same frequency points.

**Figure 11:**
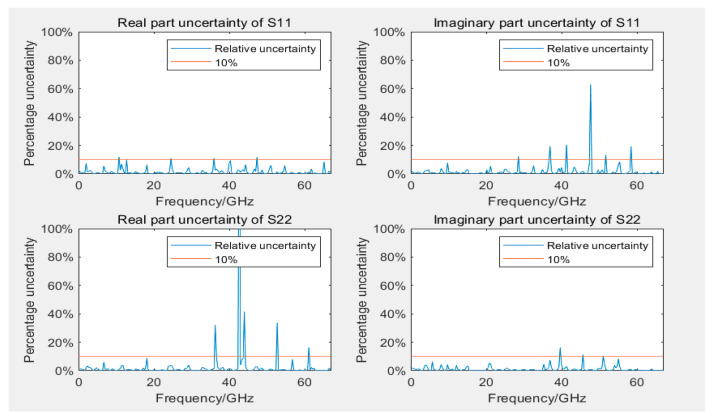
Uncertainty plots of real and imaginary parts of S11 and S22 using a 12-term error model.

**Figure 12:**
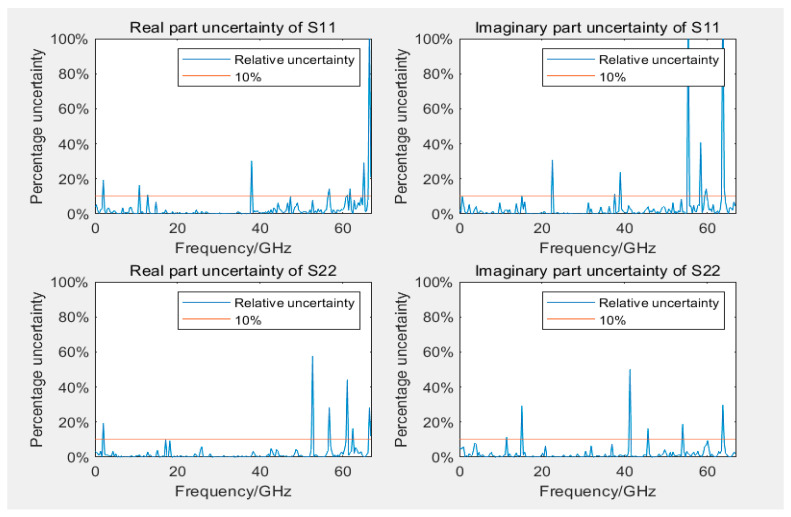
Uncertainty plots of real and imaginary parts of S11 and S22 using an 8-term error model.

**Figure 13:**
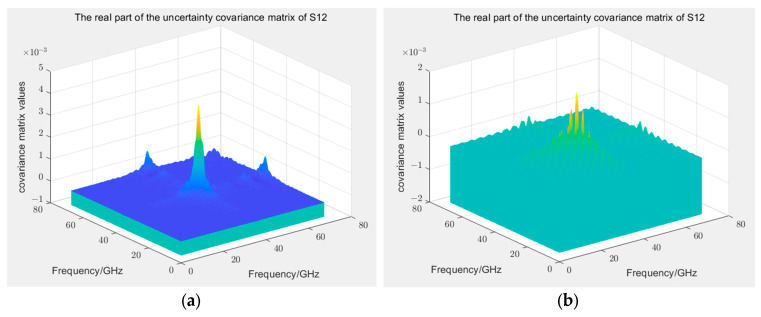
(**a**) Covariance matrix of Type A uncertainties for the real parts of S11; (**b**) Covariance matrix of Type A uncertainties for the real parts of S12.

Figure 11 and Figure 12 display the uncertainty of measuring S11 and S22 during the calibration of 12- and 8-term error models, respectively. The uncertainty of reflection coefficients was greater than transmission coefficients, with slightly more outliers because the linear measurement values of reflection coefficients were smaller, rendering them more sensitive to error models and calibration standards. Most outliers were distributed in higher frequency ranges, but some outliers occurred near 40 GHz. Similar outliers were observed in the assessment of Type A uncertainty (as displayed in Figure 13) in the same frequency range, indicating possible performance problems with the GCPW at that point. Comparing the measurement results of the two error model calibrations revealed that the results of the 12-term error model calibration were slightly better than those of the 8-error model. This phenomenon could be attributed to the 12-term error model, which has both forward and reverse error networks, partially compensating for measurement errors at the port of the 8-term error model. However, generally, the two models do not differ considerably.

When measuring with a VNA calibrated by either an 8- or a 12-term error model, the measurement performance is generally reliable across most usable VNA frequency bands. However, performance degraded near the upper bandwidth limit frequencies of the instrument and the DUT. Because of the different principles of calibration between the 8- and 12-term error models, performance differs considerably when using the same VNA and calibration standards to measure the same GCPW, which leads to slightly different uncertainty.

In subsequent studies on the uncertainty assessment of practical situations, the accuracy of the measurement results and the computational complexity of uncertainty assessment should be considered. Compared with the assessment of uncertainty using an 8-term error model, the calculation is considerably more complex and time-consuming for a 12-term error model.

Compared with Type B uncertainty, assessing Type A uncertainty is simple. In this study, after calibrating the VNA with a 12-term error model, 10 measurements were conducted on GCPW, considering the data of each measurement a 1000-dimensional random variable. Based on Equation (2), the covariance matrix of the random uncertainty can be constructed. Considering the real parts of S11 and S12 as examples, as displayed in Figure 13, with the exception of the high-frequency range, several points near the 40 GHz frequency exhibit variances reaching the magnitude of $10^{-3}$, which considerably exceeds the normal range, indicating anomalous data. However, the measurement results at most frequency points satisfy the requirements. The solution of the covariance matrix for Type A uncertainty is simple, and only a few assessment results for Type A uncertainty are provided here.

**Figure 14:**
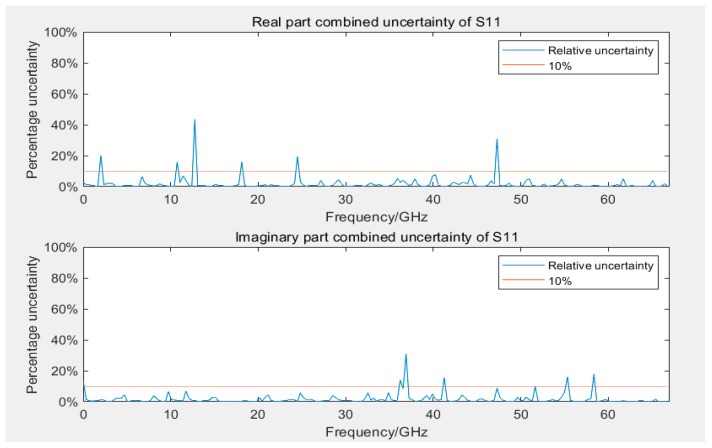
Combined uncertainty of the real and imaginary parts of S11.

**Figure 15:**
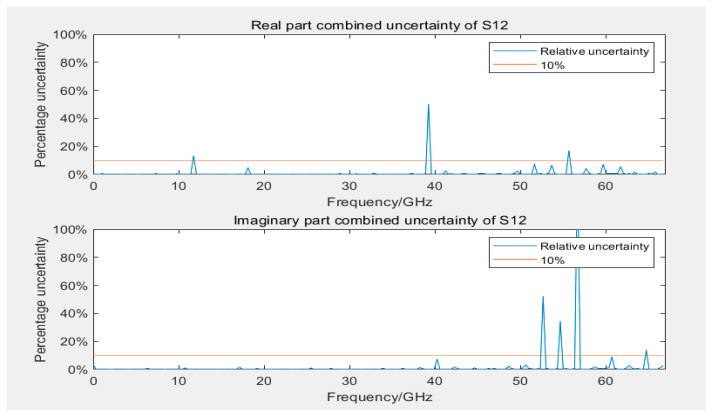
Combined uncertainty of the real and imaginary parts of S12.

After evaluating the uncertainty of Type A and Type B for the four parameters separately, the combined uncertainty can be obtained according to Equation (6), as presented in Figure 14 and Figure 15. The distribution of outliers for parameter S11 was scattered, resulting in a larger relative uncertainty. The maximum outlier for the real part of parameter S12 in combined uncertainty occurred at 40 GHz. Compared with Figure 9 and Figure 13, at this frequency point, both Type A and Type B uncertainty were considerably larger than normal values. Compared with Figure 9, the combined uncertainty included the average calculation of Type B uncertainty from multiple measurements. The uncertainty around 40 GHz near the outlier reduced considerably, which could indicate an anomaly at this frequency point in the measurement depicted in Figure 9, causing both Type A and Type B uncertainty to be abnormal. This phenomenon could be gradually eliminated by increasing the number of measurements to mitigate the effect of random errors in individual measurements.

**Figure 16:**
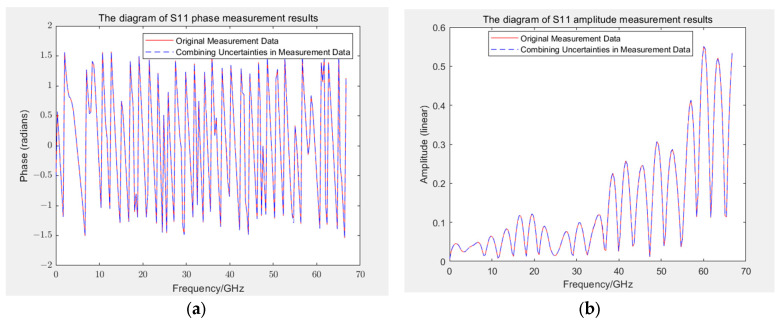
(**a**) Uncertainty superposition of the S11 phase; (**b**) uncertainty superposition of the S11 amplitude.

This study simplified intermediate-step calculations by dividing S-parameters into real and imaginary parts for uncertainty assessment. Alternatively, uncertainty can be propagated back to the amplitude and phase of S-parameters according to Equation (9), as depicted in Figure 16, to represent the combined uncertainty of the amplitude and phase of the S11 parameter. Apart from a few isolated outliers, the curve matches the original measurement values accurately. At approximately 40 GHz, where outlier frequencies occur, the relative uncertainty of the phase exceeds 20%. Compared with the uncertainty results of the real and imaginary parts, the relative uncertainties of amplitude and phase are smaller, generally between 15% and 25%, because in S-parameters, the real part is smaller than the imaginary part (or vice versa). Even if its relative uncertainty is large, then the influence of the real part on the overall amplitude and phase of the S-parameter is small.

## 6. Conclusions

When assessing the uncertainty of S-parameter measurements with a VNA, both Type B and Type A uncertainty should be considered simultaneously. Evaluating Type B uncertainty is more challenging than evaluating Type A uncertainty. Thus, in this study, we first analyzed the sources and propagation process of S-parameter uncertainty during VNA measurements. Uncertainty is stepwise propagated to the S-parameter measurement results through the Jacobian matrix. Uncertainty in the S-parameter measurements of GCPW is evaluated for 8- and 12-error models. Most Type B uncertainty is concentrated in the high-frequency range, possibly because of performance deficiencies in the self-developed GCPW. However, evaluation results for other frequency ranges are satisfactory. Several anomalies in Type A uncertainty for the 12-error model appeared near 40 GHz, whereas measurement results at other frequency points are normal. Type B uncertainty dominated the combined uncertainty, and averaging multiple measurements reduced the effect of individual measurement anomalies. Experimental results demonstrated the applicability of the proposed method for assessing the uncertainty of S-parameters.

## Figures and Tables

**Table 1:** Nominal Uncertainty (Return Loss) of Short-Circuit, Open-Circuit, and Matched Load Components.

Bandwidth	Short-Uncertainty	Open-Uncertainty	Load-Return Loss
DC~10 GHz	1.5°	2.5°	35 dB
10 GHz~20 GHz	1.5°	3.5°	34 dB
20 GHz~30 GHz	2.0°	3.5°	29 dB
30 GHz~35 GHz	2.5°	3.5°	29 dB
35 GHz~40 GHz	2.5°	3.5°	12 dB
40 GHz~50 GHz	3.5°	3.5°	12 dB
50 GHz~60 GHz	4.0°	5.0°	12 dB
60 GHz~67 GHz	4.0°	5.0°	10 dB

**Table 2:** Short-Circuit and Open-Circuit Component Circuit Parameters.

Parameters	Short-Parameters	Open-Parameters
L0/C0	1.4957 ($10^{-12}H$)	−3.5342 ($10^{-15}F$)
L1/C1	−323.18 ($10^{-24}H/Hz$)	425.24 ($10^{-27}F/Hz$)
L2/C2	11.624 ($10^{-33}H/{Hz}^{2}$)	−13.946 ($10^{-36}F/{Hz}^{2}$)
L3/C3	−0.10939 ($10^{-42}H/{Hz}^{3}$)	0.12741 ($10^{-45}F/{Hz}^{3}$)
Z0	50 (Ω)	50 (Ω)

## Data Availability

Data underlying the results presented in this paper are not publicly available at this time but may be obtained from the authors upon reasonable request.
